# The Purulent-Inflammatory Bronchogenic Cyst in the Context of a COVID-19 Infection: A Case Report

**DOI:** 10.5761/atcs.cr.24-00159

**Published:** 2025-04-01

**Authors:** Kennedy Weidner, Didier Lardinois, Mohamed Hassan

**Affiliations:** 1 Department of Thoracic Surgery, University Hospital Basel, Basel, Switzerland; 2 Department of Cardiac and Thoracic Surgery, University Hospital Magdeburg, Magdeburg, Sachsen-Anhalt, Germany

**Keywords:** bronchogenic cyst, COVID-19, superinfection, pericardial effusion, thoracotomy

## Abstract

Bronchogenic cysts (BCs) are often incidental findings during imaging and can cause compressive symptoms depending on their location and size. Infections of mediastinal BCs are serious complications that can lead to life-threatening mediastinitis. The impact of severe acute respiratory syndrome coronavirus 2 on BCs remains largely undocumented. We present a unique case of a purulent-inflammatory mediastinal BC complicated by sepsis in the context of a Coronavirus Disease 2019 infection. The Coronavirus Disease 2019 infection may result in a bacterial superinfection of the BC. However, the transmission path requires further investigation. For the surgical excision, we opted for a two-step surgical approach: thoracoscopic incision and drainage in the acute setting, followed by elective thoracotomy and resection of the BC. We confirm the safety and favorable outcome of this approach.

## Introduction

Bronchogenic cysts (BCs) are rare congenital malformations of the respiratory system, arising from abnormal budding of the tracheobronchial tree or the embryonic ventral lung bud.^1)^ These cysts are histologically lined with respiratory-type epithelium and mostly located in the mediastinum, where they account for 10%–15% of mediastinal tumors and for 50%–60% of mediastinal cystic lesions.^1)^ Additionally, BCs can be found in the lung parenchyma constituting 20%–30% of all BCs.^1)^ Typically, BCs are discovered as incidental findings during imaging, with their clinical presentation ranging from asymptomatic findings to symptoms such as hemoptysis, pneumonia, chest pain, and central venous compression due to the mediastinal mass effect.^1)^ Complications can arise from bacterial, viral, or fungal infections. However, data on how the severe acute respiratory syndrome coronavirus 2 (SARS-CoV-2) affects and complicates BCs are limited. Al Bizri et al. suggest that SARS-CoV-2 may lead to hemorrhage in intrapulmonary bronchogenic cysts (IPBCs).^2)^ In this report, we present a unique case of a purulent-inflammatory BC complicated by sepsis in the context of a Coronavirus Disease 2019 (COVID-19) infection.

## Case Report

**Fig. 1:**
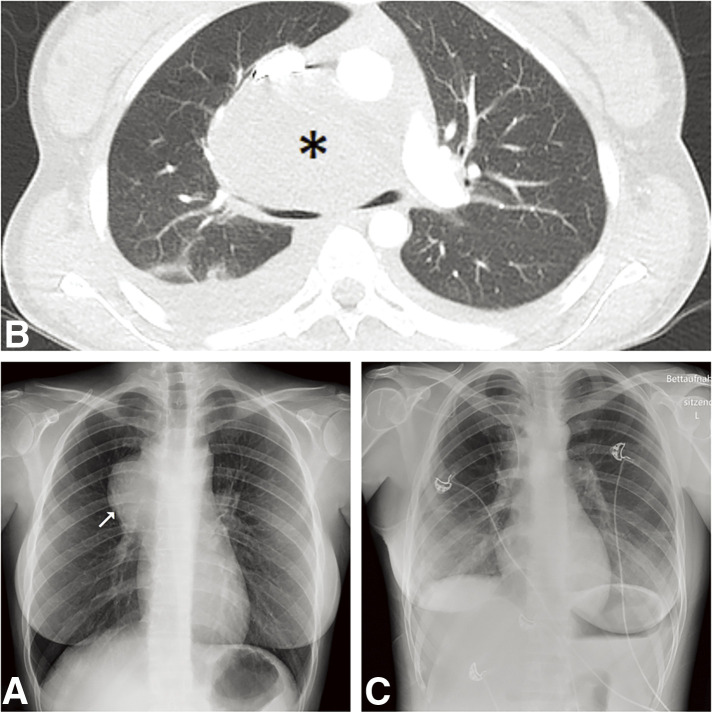
(**A**) Chest X-ray showing on the right side the bronchogenic cyst as a round mass in the anterior upper mediastinum (arrow). Differential diagnoses include lymphoma, thymoma, germ cell tumor, or an intrathoracic deeply located goiter. (**B**) CT scan showing a mediastinal mass compressing the right upper lobe artery, superior vena cava, and the main bronchi (asterisk) (**C**) Chest X-ray after the removal of the mediastinal bronchogenic cyst.

**Fig. 2:**
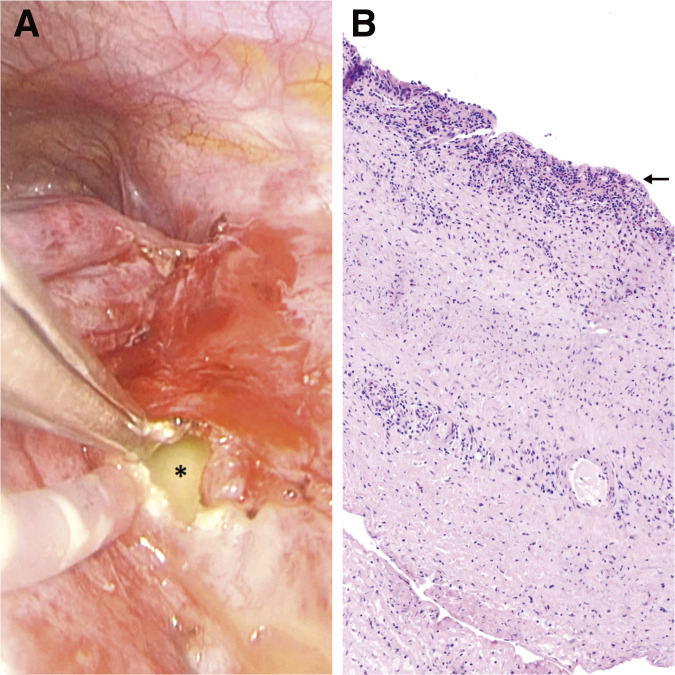
(**A**) Intraoperative image of the pus-filled bronchogenic cyst. The asterisk marks the pus. (**B**) The histological image of an epithelium-lined bronchogenic cyst (arrow; haematoxylin and eosin stain).

We present the case of a 24-year-old female patient who was transferred to our facility for further management of a mass located in the right anterior superior mediastinum. Her medical history included two doses of the mRNA COVID-19 vaccine two years prior, the use of contraceptive pills, and a diagnosis of Graves' disease, which was being treated with carbimazole. During her previous hospitalization, she presented with a cough, chest pain radiating to the back and the right arm, and subfebrile temperatures. Additionally, she complained about weight loss and extreme fatigue. Initial investigations included blood tests, blood cultures, a respiratory pathogen panel. Intravenous antibiotic therapy with co-amoxiclav (2.2 gm taken three times daily) was initiated. The laboratory results showed elevated inflammatory markers, with leukocytes at 26 G/L and a C-reactive protein level of 262 mg/L. Although blood cultures remained sterile, the respiratory panel was positive for SARS-CoV-2, with a cycle threshold value of 36.6. No infiltrates were observed in the chest X-ray, but a round mass in the right anterior upper mediastinum was detected (**Fig. 1A**). A follow-up computed tomography (CT) of the chest (**Fig. 1B**) and a positron emission tomography (PET)-CT confirmed a predominantly cystic and partially calcified mass measuring 8.2 cm in maximum diameter. The mass exhibited low metabolic activity at the periphery and caused compression of the right upper lobe artery, the superior vena cava, and the main bronchi. The clinical course was complicated by episodes of dizziness, severe hypotension, cold sweats, and cold extremities. A cardiological assessment revealed a pericardial effusion, which was effectively treated by pericardiocentesis. Neither bacteria nor malignancy were detected in the aspirate, and 2 days later the pericardial drain was removed. However, the pericardial effusion was followed by the onset of acute congestive hepatopathy. For close monitoring and further management of the mediastinal tumor, the patient was transferred to the intensive care unit (ICU) of our hospital, where antibiotic therapy was continued. The following day, we performed the surgery beginning with a thoracoscopic adhesiolysis of the right lung. Next, we exposed the mediastinal mass at the anterior upper hilum. An incision in the mass capsule, revealed purulent contents within the cavity (**Fig. 2A**). A biopsy of the capsule wall was taken for histopathological examination, and samples of purulent fluid were collected for bacterial culture. The cyst cavity was irrigated and drained, and chest tubes were placed along the mediastinum. Pathology results disclosed an epithelium-lined cyst consistent with a BC (**Fig. 2B**), with a granulocytic inflammatory cell pattern in the pus. Malignancy was ruled out, and cultures remained sterile. The operation was uneventful, and after one day in the ICU, the patient was transferred to the general ward. After 3 days in isolation due to her COVID-19 infection, she was de-isolated following a negative Polymerase Chain Reaction test for SARS-CoV-2. The patient’s health significantly improved with respiratory therapy and physiotherapy. The chest drain was successfully removed 4 days after surgery, and the patient was discharged home 2 days later with oral antibiotics, specifically co-amoxiclav 625 mg, to be taken three times a day for further 10 days. During follow-ups visits at 1 and 4 months, the patient showed no signs of inflammation or congestive hepatopathy, both clinically and in blood tests. Nonetheless, a follow-up CT and magnetic resonance imaging, revealed a persistent mediastinal BC filled with fluids. Therefore, we planned for the complete removal of the cyst 1 month later. We successfully performed a thoracotomy with the complete removal of the BC. After 1 day in the Intermediate Care Unit, the chest tube was removed and the patient was transferred to the general ward, where she recovered swiftly. The postoperative chest X-ray was unremarkable (**Fig. 1C**), and she was discharged home one week later.

## Discussion

In this case report, we present a rare case of a BC in the context of a COVID-19 infection which was discovered incidentally via imaging during a hemodynamically significant infection complicated by pericardial effusion. Infections of BCs are rare and typically occur through the same mechanisms as common respiratory infections (mainly through droplet infection and the inhalation of pathogenic microorganisms) when the cyst communicates with the tracheobronchial system or lung parenchyma.^3,4)^ In contrast, infections in non-communicating BCs are typically iatrogenic resulting from diagnostic or therapeutic interventions on the cyst, or less commonly, from other extrathoracic infections.^3,4)^

To date, there is limited data on the effects of SARS-CoV-2 on BCs. Al Bizri et al. reported a case of an anticoagulated COVID-19 patient with an IPBC, suggesting that a COVID-19 infection may lead to massive intra-cystic inflammation and secondary micro-capillary bleeding, potentially resulting in an intra-cystic hemorrhage due to anticoagulation.^2)^ It is well established that viral respiratory infections can result in secondary bacterial infections (superinfections) of the lung. However, the mechanism by which the virus can lead to a secondary bacterial infection is not well understood.^5)^

In the context of a COVID-19 infection, there is evidence that the virus impairs respiratory epithelial cells, which line most of the airways, by inhibiting mucociliary clearance. Virus-induced cell death of Type II pneumocytes, for example, reduces mucociliary clearance, increases bacterial adherence receptors, and facilitates bacterial attachment to mucins, and colonization.^5)^ In addition, COVID-19 weakens the signal transmission for neutrophil granulocytes recruitment, further enhancing bacterial adhesion to the respiratory epithelium.^5)^ Moreover, the interactions between the virus and the respiratory epithelium cells trigger the secretion of proinflammatory molecules, potentially damaging the epithelium.^5)^

Given that the BC is epithelial lined similarly to the respiratory epithelium, we hypothesize that these processes may provide an environment conducive to bacterial purulent superinfections. However, this hypothesis is limited by the fact that we did not test the cystic fluid for SARS-CoV-2 during the initial surgery, leaving the viral presence in the BC unconfirmed. Additionally, it remains unclear how respiratory pathogens are transmitted to the mediastinal BC, since radiologically the BC and the tracheobronchial tree or lung parenchyma do not communicate. Transmission of SARS-CoV-2 via blood or lymphatic routes has not been reported in the literature. Our patient also has had no history of interventions or sepsis before her admission, which can explain an iatrogenic or hematogenous infection pathway to the mediastinal BC. A theoretical explanation may be that there are communications and transmissions on a microscopic or molecular level. Alternatively, it is possible that the patient suffered from a bacterial co-infection, which, as noted by Garcia-Vidal et al. is relatively rare for community-acquired co-infections.^6)^ However, the transmission path of pathogens to radiologically non-communicating BCs of patients who have never had a history of sepsis or interventions on the cyst merits further research.

The diagnosis of the BC was followed by a hemodynamically significant pericardial effusion, though the cause of the pericardial effusion remains unclear, and no direct link to the BC has been established. Mesland et al. and also Gamrekeli et al. have documented the simultaneous occurrence of BCs and pericardial effusions.^3,7)^ According to Vakamudi et al., pericardial effusions can arise from a variety of causes, including idiopathic conditions (especially in the context of idiopathic pericarditis) and inflammatory processes such as bacterial and viral infections or autoimmune processes.^8)^ Given the presence of a COVID-19 infection, a bacterial infection in the BC, and also an autoimmune condition (Graves’ disease), it is reasonable to propose that the etiology of this patient’s pericardial effusions is multifactorial. Nevertheless, it is advisable that clinicians consider the possibility of BC infection in patients presenting with pericardial effusion.^7)^ Compression of the superior vena cava is a significant complication of mediastinal BC, and when accompanied by a pericardial effusion, it can lead to severe hemodynamic instability and congestive hepatopathy. Surgical excision of the BC remains the primary treatment.^1)^ In the context of a pus-filled BC with sepsis, we initially performed a thoracoscopic incision and drainage under adequate antibiotic therapy, followed by surgical removal of the mediastinal BC once there were no more signs of inflammation. This surgical approach was safe, and the outcome was favorable.

## Conclusion

A COVID-19 infection may lead to a bacterial superinfection of a BC. However, the transmission path of pathogens to non-communicating BCs needs further research. Patients with a mediastinal BC may present with a pericardial effusion. For a pus-filled BC, we recommend a thoracoscopic incision and drainage in the acute setting, followed by elective thoracotomy and resection of the BC. We confirm the safety and favorable outcome of this surgical approach.

## Declarations

### Ethics approval and consent to participate

Not applicable.

### Consent for publication

We have obtained the patient’s written informed consent for publication of this case report.

### Funding

This work was not funded by a grant or any other source of external funding.

### Data availability

The data supporting the results are provided within the article, and no further source data are needed.

### Author contributions

Conceptualization – Mohamed Hassan (lead), Didier Lardinois (equal), and Kennedy Weidner (supporting)

Data curation – Kennedy Weidner

Investigation – Kennedy Weidner

Methodology – Mohamed Hassan (lead), Didier Lardinois (equal), and Kennedy Weidner (equal)

Resources – Mohamed Hassan (lead), Didier Lardinois (supporting), and Kennedy Weidner (supporting)

Supervision – Mohamed Hassan (lead) and Didier Lardinois (supporting)

Visualization – Kennedy Weidner

Writing – original draft – Kennedy Weidner

Writing – review and editing – Mohamed Hassan (lead), Surgical Outcome Research Center Basel (equal), Didier Lardinois (equal), and Kennedy Weidner (equal)

Critical review and revision – Kennedy Weidner (lead), Mohamed Hassan (equal), and Didier Lardinois (equal)

Final approval of the article – Kennedy Weidner (lead), Mohamed Hassan (equal), and Didier Lardinois (equal).

### Disclosure statement

None of the authors has a financial relationship with a commercial entity that has an interest in the subject of the presented manuscript or other conflicts of interest to disclose.
